# The Selection of Lives for Insurance

**Published:** 1898-08-06

**Authors:** 


### THE SELECTION OF LIVES FOR INSURANCE.

In his opening address before the section of Medicine in Relation to Life Assurance, the president, Dr. Claud Muirhead, said that the business of life insurance had of late years made enormous advances, and that huge sums of money were now and again set aside in this way, not merely for their original humane and beneficent purpose—to secure to the widow and her children on the death of the head of the house some means by which they might be saved from the terror of actual poverty—but it was also largely used for commercial purposes. It might astonish some of those present, as it certainly amazed him, to learn that in the United Kingdom the liabilities of life assurance companies amounted to no less a sum than £263,000,000. This sum was so huge that one had difficulty in appreciating it; but it served to give some little idea of the vastness of this enterprise, and of the enormous responsibility imposed upon those who had to administer and find safe and profitable investments for those ever-growing funds. Nearly one-half of the population of the United Kingdom (exactly 43·70 per cent.)—men, women, and children of all ages—was insured in some form. Apart from the purely personal relation to life assurance, the medical profession was and had for long been associated with the companies in enabling them to make judicious selection of lives proposed for assurance. Not much before the commencement of this century were the services of a medical man asked for. On reference to the first proposals of the Scottish Widows' Fund Society in 1814, he found that the applicant had to appear before a medical man, and produce a certificate to this effect: "I do hereby certify that ——— did appear before me this day, and that I have known —— for —— years, and that to the best of my knowledge he hath never been afflicted with gout, asthma, or any disease which tends to the shortening of life, and that I do believe his present state of health to be good, and his habits of living not such as to endanger life." It would be observed that most attention was paid to the general appearance of good health, without apparently any examination of the applicant whatever. It was curious to note how concerned the directors were at that early date to ascertain if the applicant had suffered from gout. It did not appear how they dealt with a case where the man confessed to having been afflicted with gout, but evidently they were impressed with the fact that gout tended to the shortening of life—a fact which was later lost sight of, and had only recently been recognised as a condition calling for a surcharge on the premium. In 1830, these perfunctory reports were no longer considered satisfactory, and a certificate was required from the private medical attendant, to whom a series of specific questions were addressed with regard to the general state of health of the applicant, the nature of any serious illness, suspicion of any organic affection, any injury, his habits, and constitution. That was apparently the first time the society deemed it prudent to retain the services of a physician to aid them in the sifting of cases. It would thus appear that, coincident with the advance in medical science, and the spreading of the knowledge of more precise means of physical diagnosis, the companies sought for and obtained more exact information as to the actual condition of proposers before admitting them to the benefits of their societies. And so the process of selection of the most healthy lives had gone on to this day, each company trying, from time to time, to improve upon the medical forms which were placed before their examiners, in order to obtain as complete and faithful an account of the proposer as possible, both as to his family and personal history, as well as to his physical condition. There existed a popular belief that all that care was of little use, and that the benefits of selection were entirely lost after the first five or six years. Acting on that assumption, companies had been started to accept lives without any medical examination. But, so far as he was aware, they had not proved a success, as one would naturally predicate. For, to them, all the rejected of other offices would, as a matter of course, repair, and the healthy would, equally, as a matter of course, carefully shun such offices. But the fallacy of this had been repeatedly exposed, and by none more effectively than by Dr. Sprague and Mr. Meikle, of Edinburgh, who had shown that, after elimination of the voluntary withdrawals, the advantages of selection diminished at all ages with duration of the policies, rapidly at young ages, slowly at middle life, while in the older it probably never entirely disappeared. It was to the credit of the medical profession that they, by their careful examination of candidates for insurance, largely contributed the data which enabled the actuaries to formulate their tables of expectation of life for healthy males, and thus to estimate the mathematical value of the chance of man attaining his expectancy. But infinitely more difficult was the problem which was constantly submitted to them, how equitably to assess the value of under-average lives, how sufficiently to protect the societies against loss, and how, at the same time, not to over-charge such candidates for assurance. Here they had no fixed and mathematically correct data by which to guide their decisions; they had no aggregated groups of persons suffering from the same disorder, though perhaps differing greatly in form, which might supply a basis by which to adjudicate upon the special case before them. And if the extra rates recommended by medical officers had, in the past, saved the offices from loss, he was bound to admit that that happy result had been arrived at without the aid of any scientific formula, and by merely empirical methods.

